# Elevated SPARC Disrupts the Intestinal Barrier Integrity in Crohn's Disease by Interacting with OTUD4 and Activating the MYD88/NF‐κB Pathway

**DOI:** 10.1002/advs.202409419

**Published:** 2025-01-30

**Authors:** Jiayu Wang, Yuxin He, Xingchao Zhu, Jinghan Zhu, Zilin Deng, Huan Zhang, Yanjun Chen, Guangbo Zhang, Tongguo Shi, Weichang Chen

**Affiliations:** ^1^ Jiangsu Institute of Clinical Immunology The First Affiliated Hospital of Soochow University Suzhou 215000 China; ^2^ Department of Gastroenterology The First Affiliated Hospital of Soochow University Suzhou 215000 China; ^3^ Jiangsu Key Laboratory of Clinical Immunology Soochow University Suzhou 215000 China; ^4^ Infectious Disease Department The Fourth Affiliated Hospital of Soochow University Suzhou Dushu Lake Hospital Suzhou 215000 China

**Keywords:** CD, intestinal barrier, MYD88, OTUD4, SPARC

## Abstract

Disruption of the intestinal epithelial barrier results in increased permeability and is a key factor in the onset and progression of Crohn's disease (CD). The protein SPARC is primarily involved in cell interaction and migration, but its specific role in the intestinal epithelial barrier remains unclear. This study demonstrates that SPARC is significantly overexpressed in both CD patients and murine models of colitis. Furthermore, mice deficient in SPARC exhibits resistance to chemically induced colitis, a phenomenon associated with the modulation of barrier‐associated proteins. Mechanistically, it is elucidated that SPARC competitively binds to OTUD4 in conjunction with MYD88, facilitating the translocation of p65 from the cytoplasm to the nucleus and subsequent activation of the p65‐MLCK/MLC2 pathway, thereby compromising barrier integrity. Additionally, it is identified that the elevated expression of SPARC in CD is regulated via the METTL3‐YTHDF1 axis. These findings indicate that SPARC levels are elevated in patients with CD and in colitis‐induced mice, leading to intestinal barrier damage through direct interaction with OTUD4 and subsequent activation of the MYD88/p65/MLCK/MLC2 signaling pathway. Consequently, targeting SPARC or the OTUD4/MYD88/p65/MLCK/MLC2 axis may offer novel insights into the molecular mechanisms underlying CD and represent a potential therapeutic strategy.

## Graphical abstract

This study reveals that SPARC activates the MYD88/NF‐κB/MLCK/MLC2 pathway by interacting directly with OTUD4, leading to intestinal barrier damage. In addition, the METTL3‐YTHDF1 axis promotes SPARC expression. Therefore, targeting SPARC or the OTUD4/MYD88/NF‐κB/MLCK/MLC2 axis may provide new insights into the molecular basis of CD and may serve as an alternative therapeutic option.

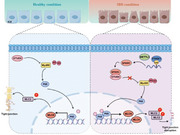

## 1 Introduction

Crohn's disease (CD) is a class of gastrointestinal inflammatory diseases whose etiology is still unclear.^[^ ^1^ ^]^ It is currently believed that genetic factors, disturbance in intestinal flora, defects in the intestinal mucosal barrier, abnormalities in mucosal immune regulation, and other factors are involved in the onset and progression of the disease.^[^ ^2^ ^]^ Among these factors, the compromised intestinal mucosal barrier plays a crucial role in the pathogenesis of CD.^[^ ^3^ ^]^

The intestinal mucosal barrier consists of a certain thickness of extracellular mucus layer, intestinal epithelial cells, and tight junctions(TJ), which play an important role in maintaining the integrity of the intestinal barrier and resisting microorganisms and exogenous antigens.^[^ ^4^, ^5^ ^]^ Structural and functional damage of the intestinal mucosal barrier, further triggers abnormally persistent immune activation, and has been implicated in the initiation and progression of CD.^[^ ^6^, ^7^ ^]^ In mice, dysfunction or deficiency of the intestinal mucosal barrier caused the development of CD symptoms or enhanced sensitivity to experimental colitis.^[^ ^8^, ^9^ ^]^ Therefore, a thorough investigation into the role of the intestinal barrier in the pathogenesis of CD will provide new targets for the treatment of CD.

Secreted protein acidic and cysteine‐rich (SPARC), a member of the extracellular matrix protein family, is widely expressed in many cell types, tissues, and organs.^[^ ^10^, ^11^ ^]^ SPARC has been implicated in various biological functions, including morphogenesis, tissue remodeling, cell migration, and proliferation.^[^ ^12^, ^13^, ^14^ ^]^ Additionally, SPARC also plays a significant role in the initiation and progression of inflammatory bowel diseases. For instance, in active ulcerative colitis patients, there is an upregulation of SPARC mRNA levels and protein expression which significantly correlates with histological activity, making it a potential marker for intestinal inflammation.^[^ ^15^ ^]^ SPARC knockout leads to significant alleviation of disease progression in a 2,4,6‐trinitrobenzene sulfonic acid (TNBS)‐induced colitis mice‐induced mouse model of enteritis by impacting the differentiation of intestinal Th17 cells.^[^ ^16^ ^]^ However, whether SPARC is involved in modulating the intestinal barrier and CD has not been determined.

In this study, we explored the role and potential molecular mechanism of SPARC in modulating intestinal barrier and CD progression. We observed that SPARC was highly expressed in CD patients and animal models of experimental colitis and was positively associated with the disease progression. We also generated SPARC knockout mice, cell lines, and colonic organoids to demonstrate that the depletion of SPARC preserved the integrity of the intestinal epithelial barrier by interacting with OTUD4 and inhibiting the MYD88/NF‐κB/MLCK pathway.

## 2 Results

### 2.1 SPARC was Highly Expressed in CD Patients and Colitis Model Mice

**Figure 1:**
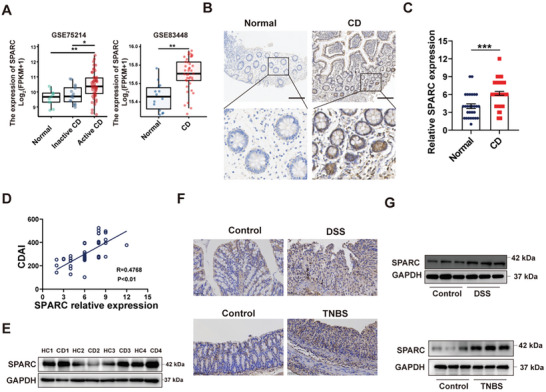
SPARC expression is elevated in the intestinal mucosa of CD patients and colitis mice A) Expression levels of SPARC in normal tissues and CD tissues based on the GSE75214 and GSE83448 datasets. B) Representative IHC images of SPARC in normal tissues and CD tissues. Scale bars, 200 µm. C) SPARC protein expression based on IHC staining index in normal tissues (n = 28) and CD samples (n = 46). D)Correlation between SPARC expression and CDAI in patients with CD (n = 46). E) The protein level of SPARC in the colonic mucosa of healthy control (HC) and CD patients was analyzed by Western blot. F) The expression of SPARC in DSS‐induced and TNBS‐induced colitis was measured via IHC. Scale bars, 50 µm. G) SPARC protein expression in DSS‐induced and TNBS‐induced colitis mice was assessed by Western blot.

First, we analyzed mucosal SPARC mRNA expression in CD patient specimens from GSE75214 and GSE83448 datasets. The results indicated that SPARC was highly expressed in CD tissues compared with specimens from normal controls (**Figure** **1**A). Furthermore, we observed that the mRNA levels of SPARC were higher in active CD patients than those in inactive CD patients from the GSE75214 database (Figure 1A). Then, IHC analysis was conducted to investigate the protein expression of SPARC in our cohort of CD patients. As shown in Figure 1B,C, the protein levels of SPARC were significantly higher in CD tissues than in normal tissues. We also assessed the association between SPARC and CD disease severity and observed a positive correlation between SPARC expression and CDAI (Figure 1D). Moreover, western blotting analysis also demonstrated a marked increase in SPARC protein in patients with CD (Figure 1E). Subsequently, we constructed DSS and TNBS‐induced colitis, two widely used chemically‐induced experimental colitis models. IHC and western blotting analysis showed that SPARC was significantly increased in the experimental group compared with the control group (Figure 1F,G).

### 2.2 SPARC Knockout Alleviated Inflammation in Experimental Colitis Mice

**Figure 2:**
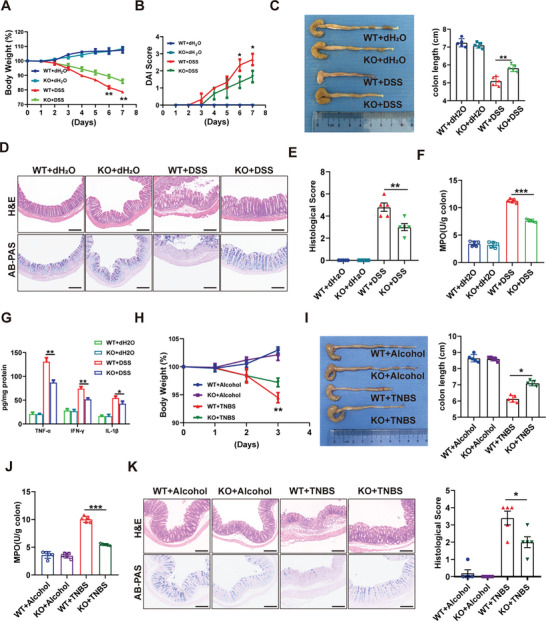
SPARC knockout resists DSS‐ and TNBS‐induced colitis. A–G) SPARC knockout and wild‐type (WT) mice were treated with 2.5% DSS dissolved in the drinking water for 7 days. Body weight loss (A), disease activity index (DAI) score (B), and colon length (C) were measured. (D) Sections of paraffin‐embedded colon tissues were stained with H&E and AB‐PAS. Scale bars, 200 µm. (E) Histological scores of the colon of colitis mice. (F) The myeloperoxidase (MPO) activity of the colon was assessed by an MPO kit. (G) Relative protein levels of TNF‐α, IFN‐γ and IL‐1β in the colon were assessed by ELISA. H‐K) SPARC knockout and WT mice were treated with 2.5% TNBS. Body weight loss H) and colon length I) were measured. J) The myeloperoxidase (MPO) activity of the colon was assessed by the MPO kit. K) Sections of paraffin‐embedded colon tissues were stained with H&E and AB‐PAS. Scale bars, 200 µm. Histological scores of the colon of colitis mice.

To systematically test the function of SPARC in CD, we generated whole‐body SPARC KO mice (Figure S1A–C, Supporting Information). Then, paired cohorts of SPARC KO and WT mice were exposed to feeding water containing DSS. Compared with WT mice, SPARC KO mice challenged with DSS lost less weight (**Figure** **2**A), showed lower DAI (Figure 2B), and had longer colon lengths (Figure 2C). Histological examination showed that DSS‐induced colitis affected all layers of the colon, including damage to the colonic epithelium and infiltration of lymphocytes in the lamina propria (Figure 2D). SPARC KO markedly improved the histological characteristics induced by DSS in mice (Figure 2E). Moreover, in comparison with WT mice, the activity of MPO, a marker of neutrophil infiltration, was significantly diminished in the colon of SPARC KO mice (Figure 2F). In addition, SPARC knockout significantly reduced the mRNA and protein expression of pro‐inflammatory cytokines including TNF‐α, IFN‐γ, and IL‐1β (Figure S2A,B, Supporting Information; Figure 2G). A similar result was found in chronic DSS‐induced colitis and TNBS‐induced colitis (Figure 2H–K; Figure S2C–G, Supporting Information). These results suggest that SPARC contributes to experimental colitis.

### 2.3 Downregulation of SPARC Preserves the Integrity of Intestinal Epithelial Cells

**Figure 3:**
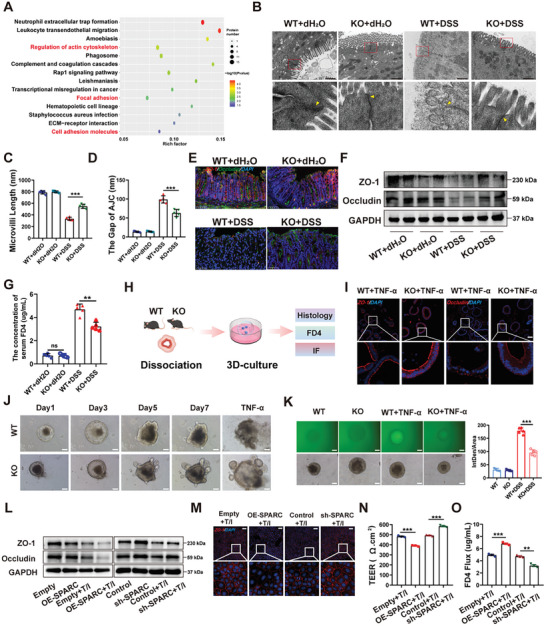
Repression of SPARC expression improves the integrity of the intestinal epithelial barrier in an inflammatory setting. A) Proteomic analysis of sorted intestinal epithelial cells from DSS colitis mice. B) Representative TEM images of the colon from DSS colitis mice, the arrows represent TJ. Scale bars, 1 µm. C,D): Microvilli length (C) and AJC space (D) of colonic epithelial cells were measured based on the TEM images. E) Representative immunofluorescence images of ZO‐1 and occludin in the colon of DSS colitis mice. Scale bars, 50 µm. F) The protein expression of ZO‐1 and occludin in the colon of DSS colitis mice was detected by western blotting. G)The concentration of serum FD4. H) Experimental design for a mouse colonic organoid model. I) Representative immunofluorescence images of ZO‐1 and occludin in mouse colonic organoids. Scale bars, 50 µm. J) Colonic organoids Intestinal crypt cells were obtained from WT and SPARC‐KO mice and organoids were observed daily. On day 7, the organoids were stimulated with mouse recombinant TNF‐α for 24 h. Scale bars, 100 µm. K) The FD4 permeability of colonic organoids from WT and SPARC‐KO mice after TNF‐α treatment was measured by confocal microscopy. Scale bars, 100 µm. The mean fluorescence intensity of FITC‐D4 was measured. L) The protein expression of ZO‐1 and occludin in Caco‐2 cells stimulated by TNF‐α/IFN‐γ. M) Representative immunofluorescence images of ZO‐1 in Caco‐2 cells stimulated by TNF‐α/IFN‐γ. Scale bars, 50 µm. N,O) TEER and FD4 permeability of Caco‐2 monolayer cell model after TNF‐α/IFN‐γ stimulation.

To investigate the potential mechanism of SPARC in the progression of colitis, proteomics analysis was performed in intestinal epithelium from WT or SPARC KO mice challenged with DSS. The KEGG pathway analysis showed that differential proteins were primarily enriched in some pathways including regulation of the actin cytoskeleton, focal adhesion, and cell adhesion processes (**Figure** **3**A). These enriched pathways were closely associated with an intestinal barrier,^[^ ^17^, ^18^, ^19^ ^]^ hinting that SPARC KO may contribute to maintaining intestinal barrier function in colitis. Further analysis by TEM revealed that compared with WT mice treated with DSS, SPARC KO mice challenged with DSS had longer and more microvilli, less damaged intracellular junction complexes, and decreased paracellular space with saccular dilatation (Figure 3B–D). The results of immunofluorescence and western blot noted that the protein levels of TJ proteins including ZO‐1 and Occludin were higher in SPARC KO mice challenged with DSS or TNBS than in WT mice treated with DSS or TNBS (Figure 3E,F; Figure S2H–J, Supporting Information). By quantifying the passage of FD4 across the epithelial barrier, we observed a significantly elevated intestinal permeability in WT mice compared to SPARC KO mice (Figure 3G; Figure S2K, Supporting Information). Furthermore, we quantified the serum levels of DAO and D‐LA, two crucial indicators of intestinal mucosal barrier damage,^[^ ^20^ ^]^ in mice challenged with DSS or TNBS. The results showed a significant reduction in DAO and D‐LA in SPARC KO mice during colitis compared with WT mice (Figure S2L, Supporting Information). To further investigate the role of SPARC in intestinal epithelial cells, an adeno‐associated virus (AAV)9 carrying a villin‐promoter‐driven short hairpin RNA (shRNA) targeting SPARC (shSPARC‐AAV) was constructed (Figure S3A, Supporting Information). Western blot results showed that shSPARC‐AAV significantly down‐regulated the expression of SPARC in colon epithelial cells (Figure S3B, Supporting Information). To explore the effect of shSPARC‐AAV on colitis and the intestinal barrier, mice were divided into three groups: the control group, DSS+AAV group, and DSS+ shSPARC‐AAV group. Compared with mice in the DSS+AAV group, mice in the DSS+ shSPARC‐AAV group displayed a decreased in weight loss, DAI, and colon length (Figure S3C–F, Supporting Information). Additionally, the depletion of SPARC in intestinal epithelial cells resulted in alleviated histological damage, characterized by lymphocyte infiltration into the lamina propria and colonic epithelial injury, in mice subjected to DSS challenge (Figure S3G–H, Supporting Information). Moreover, SPARC knockdown in intestinal epithelial cells reduced levels of inflammation‐related factors such as TNF‐α, IFN‐γ, and IL‐1β in DSS‐treated mice (Figure S3I, Supporting Information). Furthermore, compared with those in the DSS+AAV group, mice in the DSS + shSPARC‐AAV group exhibited fewer barrier impairment, evidenced by the DAO, D‐LA, and FD4 analysis as well as the expression of ZO‐1 and Occludin (Figure S3J–M, Supporting Information). We further established colonic organoids from the colonic tissues of WT and SPARC KO mice (Figure 3H). In accordance with previous results, colonic organoids have a mature columnar epithelium which contains both a stem cell niche and other differentiated cell types surrounding a single luminal compartment.^[^ ^21^ ^]^ To more accurately demonstrate the effect of SPARC on the intestinal barrier, TNF‐α was used to induce intestinal barrier damage in WT or SPARC KO colonic organoids. Colonic crypts from SPARC‐KO mice formed organoids more rapidly in vitro culture than those from WT mice did (Figure 3J). Moreover, organoids derived from WT mice exhibited abnormal cell morphology compared to those derived from SPARC‐KO mice after TNF‐α stimulation (Figure 3J). As shown in Figure 3K, compared with WT colonic organoids, SPARC KO colonic organoids exhibited decreased FD4 permeability after TNF‐α treatment. Furthermore, the results of IF staining confirmed enhanced ZO‐1 and Occludin expression in the SPARC KO colonic organoids treated with TNF‐α (Figure 3I).

To investigate the cell types that expressed SPARC, we further performed an immunofluorescence assay by labeling markers of various intestinal cell types, including the epithelial cell marker EPCAM, the fibroblast marker α‐SMA, and the lymphocyte marker CD45 and CD3. We observed that SPARC co‐localizes with intestinal epithelial cells and immune cells (Figure S4A, Supporting Information). Analysis of single‐cell sequencing data from human colonic biopsy samples (GSE134809) revealed that SPARC was expressed in epithelial cells (Figure S4B–D, Supporting Information). In order to explore the potential involvement of SPARC in regulating the epithelial barrier, we employed three specific siRNAs targeting SPARC to suppress its expression in Caco‐2 cells. RT‐qPCR analysis revealed that si‐SPARC‐3 exhibited the most potent inhibitory effect on SPARC expression within Caco‐2 cells (Figure S4E, Supporting Information). Subsequently, we utilized the sequence of si‐SPARC‐3 to construct sh‐SPARC lentivirus for stable inhibition of its expression post‐transfection into Caco‐2 cells. Additionally, we established stable SPARC‐overexpressing Caco‐2 cells (Figure S4F, Supporting Information). As shown in Figure 3L,M and Figure S3G (Supporting Information), following TNF‐a/IFN‐γ incubation, there was a significant decrease in ZO‐1 and Occludin expression in stable SPARC‐overexpressing Caco‐2 cells, whereas inhibiting the expression of SPARC led to a substantial restoration of ZO‐1 and Occludin expression. Subsequently, we assessed the effects of SPARC on barrier function in Caco‐2 monolayers by measuring TEER and FD4. The results revealed that SPARC overexpression led to increased barrier dysfunction after TNF‐α/IFN‐γ incubation, as evidenced by decreased TEER and elevated FD4 flux (Figure 3N,O). SPARC knockdown had the opposite (Figure 3N,O).

### 2.4 SPARC Directly Combined with OTUD4

**Figure 4:**
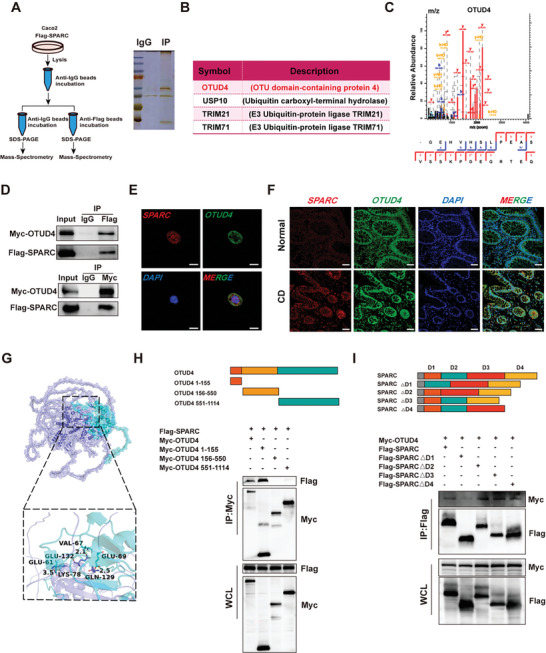
SPARC directly combined with OTUD4 A) Schematic of the purification of SPARC‐binding proteins in Caco‐2 cell using the LC‒MS/MS system. B) Four putative ubiquitin‐associated proteins interacted with SPARC were shown. C) Identification of OTUD4 protein by secondary mass spectrometry. D)Co‐IP analysis showed that Flag‐tagged SPARC interacted with Myc‐tagged OTUD4 in Caco‐2 cells. E) Immunofluorescence analysis showed colocalization of SPARC and OTUD4 in Caco‐2 cells. Scale bars, 20 µm. F) Immunofluorescence analysis showed colocalization of SPARC and OTUD4 in human colon mucosa tissues. Scale bars, 50 µm. G) OTUD4 and SPARC prediction structures were generated by Alphafold. OTUD4 was represented as a dark blue cartoon model. SPARC was shown as a cyan cartoon model. Their joint points were shown as rod structures of the corresponding colors. H) Schematic diagram of full‐length and truncated OTUD4 sequences. Co‐IP analysis showed the binding region between OTUD4 and SPARC. I) Schematic diagram of full‐length and truncated SPARC sequences. Co‐IP analysis showed the binding region between SPARC and OTUD4.

To elucidate the underlying molecular mechanism of SPARC in the regulation of the intestinal epithelial barrier during colitis, LC‐MS/MS and proteomic analysis were performed to identify potential SPARC protein interaction partners (**Figure** **4**A). Among these interaction proteins, several ubiquitin‐associated proteins, such as OTUD4, USP10, TRIM21, and TRIM71, have been identified and attracted our attention due to their important roles in the occurrence and development of diseases. Given that OTUD4, a crucial deubiquitinase, has been demonstrated to play an important role in the progression of CD,^[^ ^22^ ^]^ we selected OTUD4 as a candidate protein (Figure 4B,C). The direct interaction between SPARC and OTUD4 was verified by co‐IP (Figure 4D). Besides, the direct interaction between SPARC and OTUD4 was evidenced in the colonic tissues of DSS‐induced colitis mice (Figure S5A, Supporting Information). The co‐localization of SPARC and OTUD4 in the cytoplasm also supports their direct interaction (Figure 4E). Furthermore, SPARC and OTUD4 were observed to co‐locate in epithelial cells and immune cells in the tissue samples of CD patients (Figure 4F). Then, we predicted the binding region of SPARC and OTUD4 by Alphafold and generated a protein–protein interaction map (Figure 4G). In hydrogen bond interactions, there are multiple groups of residues between OTUD4 and SPARC for hydrogen bond formation, such as the hydrogen bond formed by LYS78 of OTUD4 and GLU61 of SPARC (Figure 4G). In order to better understand how SPARC‐OTUD4 interacts, we constructed a series of truncated fragments of SPARC and OTUD4 (Figure 4H,I). A Co‐IP analysis revealed that the D1 domain (aa 18–70) of SPARC is required for OTUD4 binding, whereas the N‐terminal domain of OTUD4 (aa 1–155) plays an important role in binding SPARC. These results suggest that SPARC directly interacted with OTUD4.

### 2.5 SPARC Disrupts the Intestinal Epithelial Barrier Through the MYD88/NF‐κB/MLCK/MLC2 Pathway

**Figure 5:**
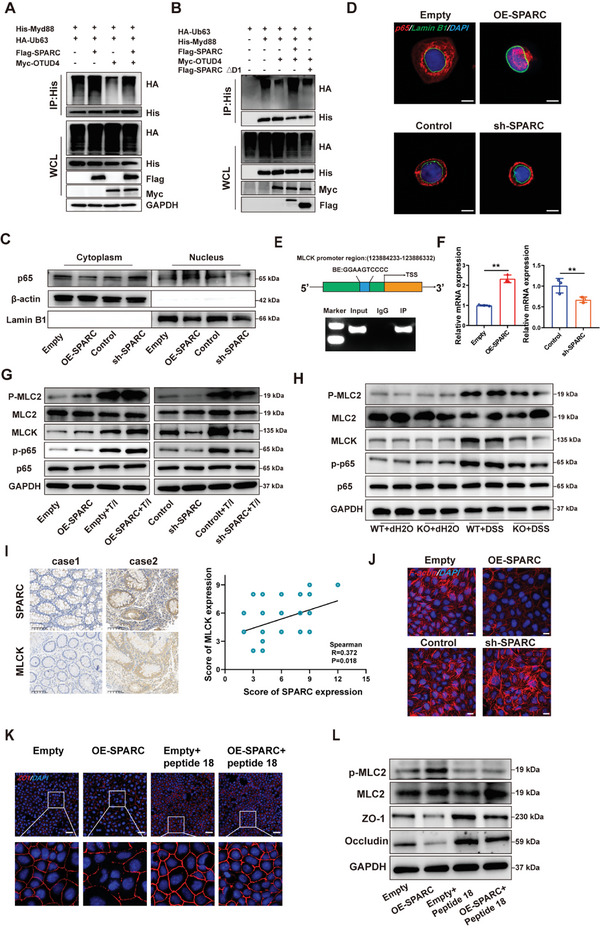
SPARC damages the intestinal epithelial barrier via the MYD88/NF‐κB/MLCK pathway A) Ubiquitination assays of MYD88 in cell lysates from HEK293T cells transfected with Flag‐SPARC vector and Myc‐OTUD4 vector. B) Ubiquitination assays of MYD88 in cell lysates from HEK293T cells transfected with Flag‐SPARC vectors with D1 domain deletion mutants and Myc‐OTUD4 vector. C‐D) Western blotting(C) and immunofluorescence(D) analysis were employed to assess the expression of p65 in the cytoplasm and nucleus of Caco‐2 cells following SPARC overexpression or knockdown. Scale bars, 10 µm. E) ChIP analysis of p65 binding to MLCK promoter in Caco‐2 cells. Normal mouse IgG was used as a control. F) The MLCK mRNA levels in SPARC overexpression or knockdown Caco‐2 cells was detected by RT‐qPCR. G) The protein expression of p65, p‐p65, MLCK, MLC2, and p‐MLC2 in SPARC knockdown or overexpression Caco‐2 cells after treatment with TNF‐α/IFN‐γ (T/I) was analyzed by western blot. H) The protein expression of p65, p‐p65, MLCK, MLC2, and p‐MLC2 in colon tissues from WT or SPARC mice challenged with DSS. I) IHC analysis of SPARC and MLCK protein expression in CD patient samples. Representative images were shown. Scale bars, 100 µm. Correlation analysis of SPARC and MLCK protein expression in human CD samples. J) The expression of F‐actin in Caco‐2 cells after SPARC knockdown or overexpression was detected by immunofluorescence. Scale bars, 20 µm. K) The expression of ZO‐1 in SPARC overexpression Caco‐2 cells treated with Peptide 18 was detected by immunofluorescence. Representative images were shown. Scale bars, 50 µm. L) The expression of ZO‐1, Occludin, MLC2 and p‐MLC2 in SPARC overexpression Caco‐2 cells treated with Peptide 18 was detected by western blot.

As shown in Figure S5B,C (Supporting Information), transfection with full‐length SPARC reversed the promotion of SPARC knockdown on ZO‐1 and Occludin expression in Caco‐2 cells after treatment with TNF‐α/IFN‐γ mixture. Moreover, full‐length SPARC overexpression abolished the effects of SPARC knockdown on TEER and FD4 flux. However, transfection with SPARC D1 domain mutants did not reverse these effects. Then, we next sought to explore how the SPARC/OTUD4 axis regulated the intestinal epithelial barrier during colitis. Previous studies have shown that OTUD4 is a K63 deubiquitination enzyme that interacts with MYD88, reduces the K63‐linked poly‐Ub levels of MYD88, and activates the MYD88‐dependent signal transduction.^[^ ^22^, ^23^ ^]^ In addition, the activation of the MYD88/NF‐κB signaling pathway was involved in the progression of IBD.^[^ ^24^, ^25^ ^]^ Therefore, we hypothesized that the SPARC/OTUD4 axis may activate the MYD88/NF‐κB signaling pathway in a K63‐polyUb‐dependent manner. The protein–protein interaction map predicted by Alphafold showed the binding region of MYD88 and OTUD4 (Figure S5D, Supporting Information). The Co‐IP results showed that MYD88 also bonds to the N‐terminal domain of OTUD4 (aa 1–155) (Figure S5E, Supporting Information). Consequently, we speculated that the interaction between SPARC and OTUD4 may inhibit the binding of MYD88 to OTUD4, thereby activating the MYD88‐dependent signal transduction. To validate this hypothesis, competitive binding co‐IP experiments were conducted, revealing a gradual decrease in MYD88 pulled down by OTUD4 as SPARC expression increased (Figure S5F, Supporting Information). This suggests that SPARC can competitively bind to OTUD4, effectively obstructing the interaction between MYD88 and OTUD4. As expected, OTUD4 overexpression significantly reduced the K63‐linked poly‐Ub levels of MYD88, and the effect of OTUD4 on MYD88‐K63 poly‐Ub was reversed by SPARC overexpression (**Figure** **5**A). Further studies revealed that SPARC lacking the D1 domain did not abolish the effect of OTUD4 on the K63‐linked poly‐Ub of MYD88 (Figure 5B). These results suggest that overexpression of SPARC disrupts the effect of OTUD4 on K63‐poly‐Ub of MYD88. Moreover, we detected the activation of NF‐κB, the downstream effectors of MYD88 signaling, in SPARC overexpression or knockdown cells. As shown in Figure 5C, overexpression of SPARC significantly increased p65 levels in the nucleus, whereas SPARC knockdown significantly decreased p65 levels. The results were also supported by immunofluorescence (Figure 5D). Overall, these data indicate that SPARC influences intestinal epithelial barrier and colitis progression by modulating MYD88/NF‐κB signaling.

As shown in Figure 3A, the KEGG analysis showed that SPARC KO influenced the actin cytoskeleton regulation in the colon of mice treated with DSS. We wondered whether SPARC‐mediated MYD88/NF‐κB signaling could regulate actin cytoskeleton‐related genes. MLCK is a component of the actin cytoskeleton^[^ ^26^ ^]^ and NF‐κB p65‐induced MLCK/MLC2 pathway activation participated in the modulation of intestinal barrier disruption.^[^ ^27^ ^]^ Hence, we inferred that SPARC contributed to intestinal epithelial barrier dysfunction in CD by regulating the OTUD4/MYD88/NF‐κB/MLCK/MLC2 pathway. The ChIP‐PCR results showed that compared with the DNA purified with control IgG, the DNA purified with the anti‐p65 antibody was significantly enriched in the predicted sequences in the MLCK promoter (Figure 5E). RT‐qPCR analysis revealed that the overexpression or inhibition of SPARC led to a corresponding increase or decrease in the mRNA levels of MLCK in Caco‐2 cells (Figure 5F). SPARC overexpression upregulated, while SPARC silencing downregulated, the protein expression of p‐p65, MLCK, and p‐MLC2 in Caco‐2 cells after treatment with TNF‐α/IFN‐γ mixture (Figure 5G). Moreover, the p‐p65, MLCK, and p‐MLC2 protein levels were markedly decreased in the colon tissues of SPARC KO mice challenged with DSS or TNBS (Figure 5H; Figure S5G, Supporting Information). Moreover, we observed that SPARC expression correlated positively with MLCK expression in our CD cohort (Figure 5I). Given that the MLCK/p‐MLC2 pathway‐mediated cytoskeletal F‐actin rearrangement was associated with intestinal epithelial barrier function,^[^ ^28^ ^]^ IF staining was performed to explore the role of SPARC in F‐actin rearrangement in Caco‐2 cells after treatment with TNF‐α/IFN‐γ mixture. The results revealed that SPARC overexpression enhanced the F‐actin rearrangement in Caco‐2 cells after treatment with a TNF‐α/IFN‐γ mixture (Figure 5J). SPARC inhibition had the opposite effect on F‐actin rearrangement in Caco‐2 cells after treatment with TNF‐α/IFN‐γ mixture (Figure 5J). Importantly, we observed that administration of MLCK inhibitor peptide 18 counteracted the inhibitory impact of SPARC overexpression on ZO‐1 and Occludin expression (Figure 5K,L; Figure S5H, Supporting Information). These results suggest that SPARC disrupts the intestinal epithelial barrier through the OTUD4/MYD88/NF‐κB/MLCK/MLC2 pathway.

### 2.6 M6A Methylation Mediates Upregulation of SPARC in CD

**Figure 6:**
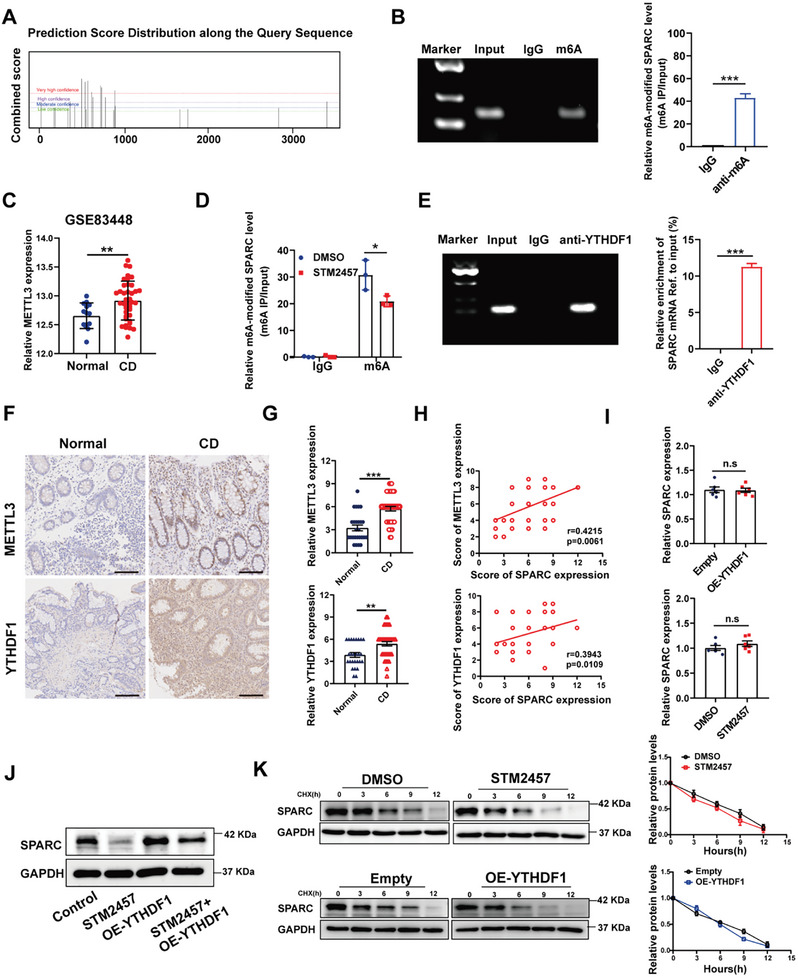
YTHDF1 promotes SPARC expression in an m6A‐dependent manner. A) SRAMP website prediction of m6A modification sites on SPARC mRNA. B) MeRIP‐PCR and agarose gel electrophoresis image showed the m6A enrichment on the mRNA of SPARC. C) Expression of METTL3 in CD tissues (n = 39) and normal tissues (n = 14) based on the GSE83448 dataset. D) The m6A levels on SPARC mRNA in Caco‐2 cells after being treated with METTL3 inhibitor STM2457. E) RIP‐qPCR and agarose gel electrophoresis image showed that YTHDF1 bonded to the SPARC mRNA. IgG was used as a negative control. F) Representative IHC images of METTL3 and YTHDF1 in normal tissues and CD tissues. Scale bars, 200 µm. G) METTL3 and YTHDF1 protein expression based on IHC staining index in normal tissues and CD samples H) IHC analysis of SPARC and METTL3 or YTHDF1 protein expression in CD patient samples. I) RT‐qPCR analysis showing the effects of METTL3 inhibition and YTHDF1 overexpression on SPARC mRNA expression in Caco‐2 cells. J) The expression of SPARC in Caco‐2 cells co‐treated with YTHDF1 overexpression vector and STM2457 was detected by western blot. K) The expression of SPARC in Caco‐2 cells treated with YTHDF1 overexpression vector or STM2457 in the presence of cycloheximide (CHX, 40 µM) for the indicated times. Quantification of the protein levels of SPARC.

N6‐ methyladenosine (m^6^A) methylation is the most common post‐transcriptional modification and is closely related to abnormal gene expression.^[^ ^29^, ^30^ ^]^ To investigate potential causes of increased SPARC in CD, we examined changes in SPARC mRNA m^6^A methylation. The SRAMP website is utilized for the prediction of m^6^A sites on SPARC mRNA (**Figure** **6**A). Subsequently, we confirmed the significant enrichment of SPARC mRNA in Caco‐2 cells by anti‐m6A antibodies using the MeRIP‐PCR method (Figure 6B). As a m6A methyltransferases, METTL3 has been reported to cause intestinal epithelial cells to pyroptosis^[^ ^31^ ^]^ and is significantly upregulated in CD patients (Figure 6C). Herein, we explored whether METTL3 was involved in the m6A methylation of SPARC. MeRIP experiments demonstrated that inhibition of METTL3 by STM2457, an inhibitor of METTL3, significantly reduced m6A modification of SPARC mRNA (Figure 6D). YTHDF1, as an m^6^A reader, plays an important role in modulating RNA metabolism including protein translation.^[^ ^32^ ^]^ The RIP experiments validated the direct interaction between SPARC mRNA and YTHDF1 protein (Figure 6E). Then, IHC analysis was performed to examine the protein expression of METTL3 and YTHDF1 in our cohort of CD patients. As illustrated in Figure 6F,G, protein levels of METTL3 and YTHDF1 were significantly elevated in CD tissues compared to normal tissues. Moreover, we observed a positive correlation between SPARC expression and METTL3 or YTHDF1 expression within our CD cohort (Figure 6H). Furthermore, inhibition of METTL3 by STM2457 reduced SPARC protein expression in Caco‐2 cells, whereas overexpression of YTHDF1 increased SPARC protein expression (Figure 6J). However, both YTHDF1 overexpression and inhibition of METTL3 had no influences on the mRNA levels of SPARC in Caco‐2 cells (Figure 6I), suggesting that YTHDF1‐mediated m6A methylation may regulate the protein translation of SPARC mRNA. In order to exclude the effect of YTHDF1 on the stability of SPARC protein, we inhibited the translation process in Caco‐2 cells using cycloheximide (CHX) and observed that SPARC protein expression was diminished in both the control group and the YTHDF1 overexpression group (Figure 6K). These findings indicate that the upregulation of SPARC protein expression induced by YTHDF1 overexpression is attributed to enhanced mRNA translation efficiency rather than increased transcription or protein stability.

## 3 Discussion

**Figure 7:**
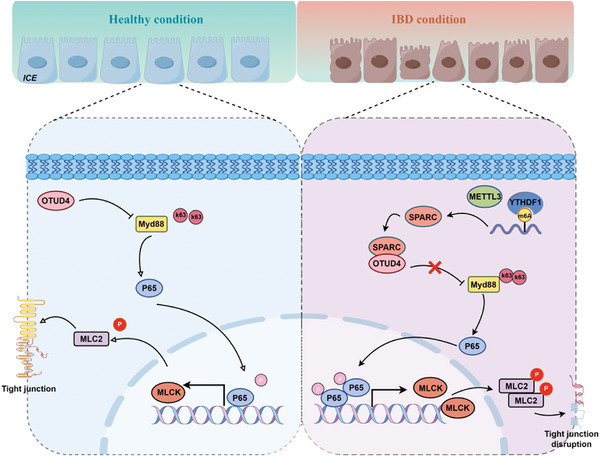
The mechanism of SPARC regulating intestinal barrier in CD. Schematic diagram of regulation mechanism in CD.

SPARC is implicated in the pathogenesis of numerous diseases, such as liver cancer, atherosclerosis, osteoarthritis, and asthma.^[^ ^10^, ^11^, ^33^, ^34^ ^]^ Elevated levels of SPARC have been observed in IBD patients and chemically induced mouse models of enteritis. However, the precise mechanism underlying its involvement in colitis remains to be fully elucidated. Our study revealed a significant increase in SPARC expression in the colonic mucosa of both CD patients and colitis mice. High expression of SPARC was positively related to disease progression. Furthermore, SPARC depletion reduced intestinal barrier damage and colitis in the mouse model, colonic organoids, and cell lines. Mechanistically, we discovered that SPARC competitively binds OTUD4 with MYD88, leading to the translocation of P65 from cytoplasm to nucleus and activation of the P65‐MLCK/MLC2 pathway, ultimately disrupting barrier integrity. Additionally, we identified that high expression of SPARC in CD is mediated by the METTL3‐YTHDF1 axis (**Figure** **7**). Previous studies showed that SPARC, an extracellular matrix glycoprotein, disrupts cell adhesion and modulates matrix metalloprotease expression.^[^ ^35^ ^]^ Considering the critical roles of cell adhesion and matrix metalloproteases in CD,^[^ ^36^, ^37^ ^]^ we hypothesize that SPARC may influence CD progression by regulating cell adhesion and the expression of matrix metalloproteases. Further investigation is warranted in future studies.

The integrity of the intestinal mucosal barrier is closely related to the pathogenesis of CD. When the intestinal mucosal barrier is damaged or compromised, it leads to increased intestinal permeability, which subsequently triggers an inflammatory response in the intestinal mucosa. TJ proteins play a crucial role in maintaining the intestinal mucosal barrier. Zhou et al., demonstrated that the downregulation of TMIGD1 exacerbates intestinal barrier impairment through the NF‐κB signaling pathway, thereby accelerating the progression of CD.^[^ ^38^ ^]^ Furthermore, dietary mannose has been shown to facilitate the repair of TJ, consequently mitigating intestinal inflammation in IL‐10 KO mice.^[^ ^39^ ^]^ In a study by Alkabie Samir et al., SPARC disrupted the barrier by downregulating the expression of ZO‐1, thus increasing endothelial cell permeability.^[^ ^40^ ^]^ This is consistent with our findings, where we observed that in vivo knockout of SPARC could prevent the increase in colonic permeability and loss of TJ integrity induced by DSS or TNBS. Besides, our in vitro data showed that knockdown of SPARC alleviated the disruption of TJs following TNF‐α/IFN‐γ treatment, resulting in reduced FITC‐dextran flux; conversely, overexpression of SPARC exacerbated the degradation of TJ proteins after TNF‐α/IFN‐γ stimulation. Therefore, under inflammatory conditions, SPARC mainly aggravates intestinal mucosal barrier damage by promoting the degradation of TJ proteins, thus confirming that SPARC can disrupt the intestinal barrier to promote CD progression.

Previous studies have reported that the intestinal mucosal barrier is also regulated by multiple factors, such as gut microbiota, cytokines, and intestinal epithelial cell apotosis.^[^ ^41^, ^42^ ^]^ Among these factors, the roles of epithelial cell apoptosis in the impairment of the intestinal epithelial barrier have received a great deal of attention.^[^ ^42^, ^43^ ^]^ Therefore, we also investigated the impact of SPARC intervention on TNF‐α/IFN‐γ induced epithelial apoptosis. The findings revealed that upregulation of SPARC significantly enhanced the apoptosis of intestinal epithelial cells following TNF‐a/IFN‐γ exposure; while downregulation of SPARC notably inhibited the apoptosis of intestinal epithelial cells after TNF‐a/IFN‐γ incubation (Figure S6A,B, Supporting Information). Consistent with in vitro results, Tunel staining demonstrated a higher rate of apoptotic cells in the colon tissues from WT mice treated with DSS or TNBS compared to SPARC‐KO mice (Figure S6C,D, Supporting Information). Taken together, these data suggest that SPARC compromises the integrity of the epithelial barrier both in vivo and in vitro. However, the mechanisms of SPARC in modulating epithelial apoptosis need to be further investigated. Moreover, we could not exclude the possibility that SPARC may influence the intestinal mucosal barrier by regulating other factors including gut microbiota and cytokines.

Deubiquitination is a key regulatory step in the ubiquitin‐proteasome system, where ubiquitination and deubiquitination processes regulate various cellular functions, such as protein degradation, cell cycle, and signal transduction, by adding or removing ubiquitin molecules.^[^ ^44^, ^45^ ^]^ OTUD4 is an enzyme involved in the deubiquitination process and is one of the deubiquitinases (DUBs) belonging to the OTU (ovarian tumor) family. In our study, we found that SPARC existed the direct interaction with OTUD4. Zhao et al., reported that OTUD4 interacted with MYD88, removing its K63‐ubiquitin modification and activating the MYD88 pathway.^[^ ^23^ ^]^ In this study, we found that the N‐terminal domain of OTUD4 (aa 1–155) is required for SPARC and MYD88. Therefore, we proposed a new molecular model where SPARC competed with MYD88 for binding to OTUD4, thereby inhibiting its catalytic deubiquitination of MYD88, leading to the increase in the K63‐linked poly‐Ub levels of MYD88 and activation of MYD88. Given that our results of LC‐MS/MS and proteomic analysis identified several potential SPARC protein interaction partners, we inferred that SPARC may regulate the intestinal mucosal barrier during CD via other interaction partners. Further investigation is needed.

The MYD88‐NF‐κB pathway plays an important role in the pathogenesis of IBD. MYD88 is a key downstream protein in Toll‐like receptor and interleukin‐1 receptor signaling, promoting inflammatory responses by activating the NF‐κB signaling pathway. Ye et al., demonstrated that dexmedetomidine alleviated intestinal barrier dysfunction and inflammation in a mouse model of ulcerative colitis by inhibiting the TLR4/MYD88/NF‐κB signaling pathway.^[^ ^46^ ^]^ It is well known that the NF‐κB‐MLCK/MLC2 pathway plays a crucial role in regulating intestinal barrier integrity. Acanthopanax senticosus polysaccharides (ASPS) inhibited the NF‐κB/MLCK pathway, thereby improving intestinal epithelial dysfunction during endotoxemia.^[^ ^47^ ^]^ Activated NF‐κB p65 translocates from the cytoplasm to the nucleus, binds to the MLCK promoter, and upregulates MLCK protein expression. During the development of IBD, MLCK‐mediated phosphorylation of MLC2 leads to TJ disruption and intestinal mucosal barrier damage.^[^ ^48^, ^49^ ^]^ This is consistent with our findings, where we observed that overexpression of SPARC promoted protein expression of p‐p65, MLCK, and p‐MLC2 in vitro and in vivo. Importantly, treatment with peptide 18, an MLCK inhibitor, reversed the effect of SPARC on intestinal mucosal barrier damage. These results suggest that SPARC/OTUD4 can activate the NF‐κB‐MLCK‐MLC2 pathway, leading to TJ disruption.

We were also concerned about the high expression of SPARC in CD and were interested in understanding the mechanism that mediated its high expression. M6A methylation is the most common post‐transcriptional modification in mammalian mRNA and plays a key role in gene expression regulation. Biologically, m6A modification is mediated by the coordinated action of methyltransferases, demethylases, and m6A‐binding proteins. In recent years, the role of m6A methylation in IBD has received increasing attention. Zong et al., showed that the m6A‐binding protein YTHDF1 regulated the translation of TRAF6 and mediated intestinal immune responses.^[^ ^50^ ^]^ Moreover, Ma et al., explored the role of the m6A demethylase FTO in UC, revealing its key role in regulating inflammation and immune responses.^[^ ^51^ ^]^ Previous studies have shown that METTL3/YTHDF1‐mediated m6A methylation plays a key role in inflammation regulation.^[^ ^52^, ^53^, ^54^ ^]^ For example, METTL3 overexpression aggravates LPS‐induced cellular inflammation in mouse intestinal epithelial cells and DSS‐induced IBD in mice.^[^ ^52^ ^]^ The specific inhibition of YTHDF1 with salvianolic acid can alleviate intestinal inflammation.^[^ ^55^ ^]^ Therefore, we inferred that the upregulation of SPARC induced by m6A methylation is associated with intestinal inflammation. In our study, we discovered that the mRNA of SPARC could be modified by m6A, which is mediated by METTL3 and YTHDF1. Moreover, we found that the METTL3‐YTHDF1 axis promoted the translation of SPARC mRNA. Our results were consistent with a previous study, which showed that YTHDF1 interacted with translation initiation factors and mediated the translation of m6A‐modified transcripts.^[^ ^32^ ^]^ However, further research is needed to confirm that the METTL3‐YTHDF1 axis promotes the translation of SPARC mRNA. Notably, the SPARC expression was also modulated at the transcriptional level in colitis. USP22 deficiency increased H3K27ac and H2Bub1 occupancy on the SPARC gene body and raised SPARC levels in mouse colons with colitis and inflammation‐related colorectal cancer.^[^ ^56^ ^]^ This finding suggests that the expression of SPARC was regulated at transcriptional and post‐transcriptional levels in colitis.

The limited sample size in this study may introduce potential statistical bias. Further investigations are needed in a larger population. Additionally, while we focused on SPARC's role in the epithelial barrier, further investigation is needed to determine whether SPARC may also influence CD progression through other mechanisms, such as neutrophil pathways. Moreover, our animal model may not fully represent the pathogenesis of CD. In future studies, we plan to employ chronic animal models to better mimic CD and validate our conclusions.

In conclusion, our findings revealed that SPARC, increased in CD patients and colitis mice, resulted in intestinal barrier damage by directly interacting with OTUD4, and activating the MYD88/NF‐κB/MLCK/MLC2 pathway. Therefore, targeting SPARC or OTUD4/MYD88/NF‐κB/MLCK/MLC2 axis may provide new insights into the molecular basis of CD and serve as an alternative treatment option.

## 4 Experimental Section

### Microarray Data and Online Website Analysis

Microarray gene expression datasets GSE75214 and GSE83448 were obtained from the GEO database (https://www.ncbi.nlm.nih.gov/geo/) and downloaded in the MINiML format. Signal cell datasets GSE134809 were obtained from the GEO database (https://www.ncbi.nlm.nih.gov/geo/) and downloaded in the 10X Genomics format. Molecular docking was predicted using AlphaFold (https://www.alphafold.ebi.ac.uk/). To ensure the accuracy of the docking results, the two protein structures were manually optimized, including water removal and hydrogenation, using AutoDockTools‐1.5.7. Protein–protein docking was then performed using the GRAMM docking server. The resulting protein–protein complex was further optimized with AutoDockTools‐1.5.7 for manual water removal, hydrogenation, and other adjustments. Finally, PyMOL was used to predict protein interactions and generate a protein interaction map.

### Human Samples

The experiments involved in human samples were approved by the Institutional Committee of the Hospital Ethics Committee of the First Affiliated Hospital of Soochow University (Suzhou, China) and informed consent was also obtained from each participant (reference number:2024‐371). The intestinal endoscopic biopsy specimens of CD patients or healthy controls were obtained from the Department of Gastroenterology, the First Affiliated Hospital of Soochow University. The specimens were used for immunohistochemistry (IHC) and Western blot assays. The Crohn's disease activity index (CDAI) was performed according to previous protocols.^[^ ^57^ ^]^

### Immunohistochemistry (IHC)

The IHC staining procedure followed standard protocols described in a previous study.^[^ ^58^ ^]^ In brief, 5 µm‐thick sections of paraffin‐embedded tissue were dewaxed, antigen‐retrieved at high temperature, blocked with non‐specific antigens, and stained with anti‐SPARC (Proteintech, Wuhan, China, #66426‐1‐Ig), anti‐MLCK (Proteintech, #21642‐1‐AP), anti‐METTL3(Proteintech, #15073‐1‐AP), and anti‐YTHDF1 (Proteintech, #66745‐1‐Ig) overnight at 4 °C. Subsequently, the sections were treated with HRP‐conjugated secondary antibody and developed using diaminobenzidine (DAB) solution. The stained slides were then examined under a microscope for evaluation of staining intensity (0, negative; 1, weak; 2, medium; 3, strong) and staining ratio (1,<25%; 2,25‐50%; 3,50‐75%; 4>75%).

### Western Blotting

Total protein was isolated from tissues or cells using cell lysate (Beyotime, Shanghai, China, #P0013) containing protease and phosphatase inhibitors. Nuclear protein and cytoplasmic protein extraction kits (Beyotime, #P0027) were used to extract cell components. β‐actin (Proteintech, #66009‐1‐Ig) was used as cytoplasmic control and Lamin B1(Proteintech, #12987‐1‐AP) was used as nuclear control. The protein concentration was measured using a BCA protein assay kit (Beyotime). Primary antibodies specific to SPARC (1:1000, CST, #8725), ZO‐1 (1:10000, Proteintech, #21773‐1‐AP), Occludin(1:10000, Proteintech, #66378‐1‐Ig), OTUD4 (1:1000, Proteintech, #25070‐1‐AP), P65 (1:1000, CST, #8242), p‐P65 (1:1000, CST, #3033), MLCK(1:1000, AiFang, #AFW8041), MLC2(1:2000, Proteintech, #10906‐1‐AP), p‐MLC2(1:1000, CST, #3674), β‐actin(1:20000, Proteintech, #66009‐1‐Ig), Lamin B1(1:2000, Proteintech, #12987‐1‐AP), and GAPDH (1:20000, Proteintech, #60004‐1‐Ig) were used. Lastly, the signal was detected by using an Enhanced Chemiluminescence (ECL) system according to the manufacturer's instructions.

### Mice and Colitis Models

All animal experiments were approved by the Animal Ethics Committee of Soochow University (reference number: 202301A0227). C57BL/6 wild type (WT) and SPARC knock‐out (SPARC^−/−^) mice were obtained from Cyagen laboratories (Cat. #KOCMP‐20692‐SPARC) and were maintained under specific pathogen‐free conditions (SPF). To establish Dextran sulfate sodium (DSS; MeilunBio, Dalian, China, #9011‐18‐1)‐induced colitis, male mice aged 6–8 weeks were given 2.5% DSS through drinking water for 7 days. The control mice were fed with sterile ddH2O. The chronic DSS colitis model was also constructed following the methods of Yu et al.^[^ ^59^ ^]^ To investigate SPARC's roles in intestinal epithelial cells, an AAV9 carrying a villin‐promoter‐driven shRNA targeting SPARC (shSPARC‐AAV) was constructed. To demonstrate the interference efficiency of shSPARC‐AAV in vivo, mice were given either shSPARC‐AAV (1 × 10^11^ VG) or control AAV (control‐AAV) for two weeks. For TNBS (Sigma–Aldrich, #2508‐19‐2)‐induced colitis, cutaneous sensitization to TNBS was induced in mice by application of 1% (wt/vol) TNBS presensitization solution for 1 week. Then, these mice were lightly anesthetized with 100 µl 2.5% (wt vol^−1^) TNBS through a catheter inserted 4 cm into the colonic lumen. Control mice received a 100 µl dose of 50% ethanol. The mice were monitored daily for weight loss, diarrhea, and bleeding to calculate the Disease Activity Index (DAI). Following euthanasia, colon tissues were fixed and embedded in paraffin for subsequent hematoxylin‐eosin and AB‐PAS staining. Neutrophil quantification was performed semi‐quantitatively by measuring myeloperoxidase (MPO) activity, utilizing the MPO Assay Kit (Jiancheng BioEngineering, #A044‐1‐1) in accordance with the manufacturer's protocols. MPO activity was determined by measuring absorbance at 460 nm.

### Genotype Identification

The genotype of SPARC^−/−^ mice was identified using a genotypic rapid identification kit (Beyotime, #D7283S) following the protocol provided by the manufacturer. Briefly, 1–2 mm of the mouse tail tip was cut and placed into a labeled centrifuge tube. DNA extraction was performed according to the instructions, and a PCR reaction was designed using specific primers to amplify the target gene region. The PCR amplification products were detected using agarose gel electrophoresis, and the genotypes were determined based on the electrophoresis results. The primers are listed in Table S1 (Supporting Information).

### RNA Isolation and RT‐qPCR

Total RNA was extracted from cells using an RNA‐Quick Purification Kit (YISHAN, China, #ES‐RN001) in accordance with the manufacturer's instructions. cDNA was synthesized using the HiScript III RT SuperMix Kit (Vazyme, China, #R323) using total RNA (1 µg) from each sample. The SYBGreen method was used to detect target genes by RT‐qPCR. The mRNA levels in each sample using GAPDH were normalized to determine relative changes in transcript numbers. The genes‐specific primer sequences can be found in Table S2 (Supporting Information).

### Transmission Electron Microscopy

The colon tissue was placed in an electron microscope fixative solution (servicebio, China, #G1102) at 4 °C and fixed for 2–4 h. The tissue was then embedded in a 1% agarose solution and fixed with 1% osmium tetroxide at room temperature for 2 h in the dark. The tissue was dehydrated using a gradient alcohol series at room temperature, embedded in epoxy resin and sectioned to a thickness of 60–80 nm using an ultra‐microtome (PT‐PC). The sections were then mounted on a 150‐mesh copper grid. The copper grid was stained with a 2% uranyl acetate saturated alcohol solution in the dark for 8 min, followed by three washes with 70% alcohol, three washes with ultrapure water, and stained with 2.6% lead citrate solution for 8 min. The grid was then cleaned three times with ultrapure water and gently blotted with filter paper. The copper grid sections were placed in a grid box and allowed to dry overnight at room temperature. The images were analyzed using a transmission electron microscope (HT7800).

### Cell Culture

The human colonic epithelial adenocarcinoma cell line (Caco‐2) and HEK293T were purchased from American Type Culture Collection (ATCC, USA) were cultured in 1640 (Eallbio, Beijing, China, #A001) containing 10% fetal bovine serum (FBS, Eallbio, #03.A16001DC) and 1% penicillin‐streptomycin (Beyotime, #C0222) in a humidified incubator with 5% CO_2_ at 37 °C.

### Cell Transfection and Infection

One control siRNA and three nonoverlapping SPARC‐specific siRNA (siRNA‐1, ‐2, and ‐3) were purchased from RiboBio (Guangzhou, China). The target sequence of siRNA are provided in Table S3 (Supporting Information).

Caco‐2 cells were cultured in 6‐well plates and transfected with SPARC‐specific siRNA (siRNA‐1, ‐2, and ‐3) and siRNA‐NC using Lipo8000 (Beyotime, #C0533) following the manufacturer's instructions. Real‐time quantitative PCR (RT‐qPCR) was used to verify the knockdown effect. Lentiviral vectors containing short hairpin RNA (shRNA) with SPARC siRNA‐3 sequence or SPARC plasmid came from GenePharma (Suzhou, China). Lentiviral particles were infected into Caco‐2 cells at an appropriate MOI (100) in a complete culture medium containing 10 µg mL^−1^ polybrene. After 24 h, the medium containing lentivirus was replaced with a fresh complete medium. The transduced cells were then cultured in a medium supplemented with purinomycin for at least two weeks. Total RNA and protein were subsequently extracted to verify the overexpression or knockdown efficiency of SPARC.

### Plasmid Construction and Transfection

The pLV2‐SPARC‐3×Flag (Flag‐SPARC), pLV2‐CMV‐OTUD4‐3×Myc (Myc‐OTUD4), and pCMV‐MYD88‐6×His (His‐MYD88) plasmids were obtained from Miaolingbio (Wuhan, China). Additionally, plasmids encoding SPARC lacking specific regions (Δ1‐Δ4) were also sourced from Miaolingbio. These truncated sequences correspond to the following amino acid residues of the SPARC protein: full length (1‐303), Δ1 (18‐70), Δ2 (71‐93), Δ3 (96‐149), and Δ4 (153‐289). The OTUD4 plasmid was similarly truncated and divided into three segments, representing amino acid sequences 1–155, 156–550, and 551–1114, respectively. Ubiquitin mutants (K63) were created through site‐directed mutagenesis, using a plasmid containing HA epitope‐tagged ubiquitin (HA‐Ub) as a template, following the Mut Express II Fast Mutagenesis Kit V2 protocol from Vazyme (Nanjing, China). Detailed information about the plasmid profiles of plasmids is shown in Figure S7 (Supporting Information). Plasmid transfections were conducted using the Lipo8000 transfection reagent, adhering strictly to the manufacturer's instructions.

### Organoid Isolation and Culture

The isolation and culture of colonic crypts in mice were previously described.^[^ ^60^ ^]^ In brief, the mouse colons were longitudinally cut and rinsed with pre‐cooled Dulbecco's Phosphate‐Buffered Saline (DPBS). They were then cut into 2 mm pieces and incubated with 2 mM EDTA at 4 °C for 30 min. The crypts were collected using a 70 µm cell filter, embedded in a matrix gel, and cultured in a mouse organoid medium. The medium was changed every three days and organoids were observed every day. In accordance with the recommendations of reagent manufacturers, various media were employed to construct organoids (Biogenous, #K2204‐MC) for diverse experimental purposes. The differentiation medium was utilized to observe the morphology of organoids. When evaluating permeability, an expansion medium was utilized.

After 7 days of culture, 100 ng mL^−1^ mouse recombinant TNF‐α (Peprotech, China, #315‐01A‐1MG) was used to stimulate organoids for 24 h. As described by Xu et al., paracellular permeability was assessed by co‐incubating organoids with 1 mg mL^−1^ FITC‐D4 (Sigma–Aldrich, #60842‐46‐8) at 37 °C for 24 h. The permeation of FITC‐D4 from the basal to the luminal side of the organoids was quantified using confocal microscopy and analyzed with ImageJ software.^[^ ^61^ ^]^

### Immunofluorescence Staining

For tight junction staining, Caco‐2 cells from different groups were seeded into a 24‐well plate at a density of 5 × 10⁴cells per well. The medium was refreshed every two days, and the cells were cultured for 21 days to ensure full differentiation and formation of a polarized monolayer structure. Afterward, the cells were treated with 100 ng mL^−1^ TNF‐α/IFN‐γ (Peprotech, #300‐01A‐100UG, #300‐02‐100UG) for 24 h, followed by immunofluorescence staining. Frozen sections of mice colon or colonic organoids were fixed with 4% PFA for 30 min and blocked with 5% BSA. Subsequently, they were incubated with ZO‐1 (1:200, Proteintech) and Occludin (1:200, Proteintech) antibodies overnight at 4 °C, followed by incubation with secondary antibodies coupled to Cy3 (Beyotime, 1:500, #A0521) or Alexa‐488 (Beyotime, 1:500, #A0423) for 1 h. Afterward, the sections were stained with DAPI (Beyotime, #P0131) at room temperature for 5 min and observed under a confocal microscope.

### Liquid Chromatography‐Mass Spectrometry (LC‒MS/MS)

Caco‐2 cells were transfected with Flag‐SPARC plasmids and lysed using an IP lysis buffer. Immunoprecipitation was carried out employing anti‐FLAG magnetic beads (Beyotime, #P2115). Following washing with IP lysis buffer 4 times, the eluted proteins were separated by SDS‐PAGE and stained with Coomassie brilliant blue. The protein bands were subsequently excised from the gel for reduction and trypsin digestion. Peptide sequence analysis was performed using a Q mass spectrometer (Thermo Fisher Scientific, USA) at Suzhou Meixin Biological Technology Co., Ltd (Suzhou, China). Protein identification was conducted using MaxQuant 1.5.5.1 software and compared against the UniProt database.

### Coimmunoprecipitation (Co‐IP) Assay

To investigate the interaction between SPARC and OTUD4 proteins, Caco‐2 cells co‐transfected with the SPARC‐Flag plasmids and OTUD4‐Myc plasmids (MiaolingBio) were harvested using IP lysis buffer. Equal amounts of cell lysates were incubated with magnetic beads overnight at 4 °C. Subsequently, the beads were isolated using a magnetic separator and washed with Tris‐buffered saline (TBS, Beyotime, #ST661) 5 times. Proteins bound to the magnetic beads were eluted by heating in SDS‐PAGE loading buffer at 95 °C for 5 min. The elution samples were then analyzed using western blotting.

### Enzyme‐Linked Immunosorbent Assay (ELISA)

Serum samples from colitis mice were collected and analyzed for diamine oxidase (DAO)(#MM‐0228M2), D‐lactic acid (D‐LA)(#MM‐43853M2), TNF‐α (#MM‐0 132M1), IFN‐γ (#MM‐0 182M1), and IL‐1β (#MM‐0040M1) using ELISA (Meimian, China) in accordance with the manufacturer's instructions.

### Evaluation of the Intestinal Epithelial Monolayer Cell Barrier and Paracellular Permeability

To establish a monolayer cell model in vitro, Caco‐2 cells stably transfected with various vectors were seeded at a density of 1 × 10^4^ cells per well in the upper chamber of a 24‐well plate (NEST, China) and cultured for ≈21 days. Changes in transepithelial electrical resistance (TEER) were assessed using an epithelial voltage resistance meter (Jingong Hongtai, China). TEER was calculated as follows:

(1) $$\mathrm{TEER}=\left(\mathrm{Total}\;\mathrm{resistance}\;-\mathrm{Blank}\;\mathrm{resistance}\right)\times \mathrm{Area}$$

For the FD4 permeability assay, the FD4 powder was mixed with Opti‐MEM (Gibco, USA) to make a 2 mg mL^−1^ solution. The FD4 solution was placed in the upper transpore chamber while the Opti‐MEM solution was placed in the lower transpore chamber. After 4 h, the fluorescence intensity of the lower chamber was measured by a TECAN Infinite F500 microplate reader. For mice, the mice were fasted for 6 h on the final day of the experiment and administered FD4 via oral gavage at a dose of 400 mg kg^−1^. 4 h post‐gavage, blood was collected from the retro‐orbital sinus, shielded from light, and centrifuged to isolate serum. Fluorescence intensity was subsequently measured with a microplate reader.

### MeRIP Assays

To perform MeRIP assays, m6A RNA enrichment kits (Epigentek, USA, #P‐9018) were utilized. The process involved using beads to specifically pull down RNA fragments containing the m6A modification. Following this, the enriched RNA was subjected to purification and elution steps to isolate the target fragments. Finally, RT‐qPCR was employed to accurately quantify the methylation changes in the target genes. The primers are listed in Table S1 (Supporting Information).

### RNA Binding Protein Immunoprecipitation (RIP)

RIP was performed using a RIP kit (BersinBio, Guangzhou, China, #Bes5101) according to the manufacturer's protocol. Cells were cleaved on ice using RIP lysis buffer. After incubation with magnetic protein A/G beads and 5 µg of normal IgG or YTHDF1 antibodies overnight at 4 °C, RNA‐protein complexes were continuously washed with eluent and treated with protease K at 55 °C for 1 h. The binding RNA was extracted for RT‐qPCR analysis. The primers are listed in Table S2 (Supporting Information).

### Chromatin Immunoprecipitation (ChIP)

A ChIP experiment was performed as described previously.^[^ ^62^ ^]^ Briefly, cells were crosslinked with 1% formaldehyde, cleaved with a cracking solution, and their genomic DNA was processed by ultrasound. After incubation with an anti‐p65 antibody (Proteintech, #66535‐1‐Ig) or control IgG overnight at 4 °C, DNA fragments were mixed with protein G Agarose. Then, the complexes of antibody, antigen, and DNA fragments were collected and eluted with buffer. The short hairpin DNA was detected by RT‐qPCR and DNA agarose gel electrophoresis. The primer sequences for the MLCK promoter region were listed below: Forward, 5′‐CAAAGTGTCCCTCAAAGTGTC‐3′ Reverse, 5′‐TCACCCAGCCTCAGGTATTT‐3′.

### Cell Apoptosis Analysis

The cell apoptosis experiment was conducted as previously described.^[^ ^63^ ^]^ Briefly, Caco‐2 cells cultured in 6‐well plates were harvested and stained with Annexin V‐PE and 7‐AAD (BD Biosciences, USA, #556547), followed by incubation at room temperature in the dark for 20 min according to the manufacturer's instructions. Subsequently, apoptosis was promptly analyzed using flow cytometry (Beckman Coulter, USA). FlowJo software was utilized for quantifying the apoptosis rate.

### TUNEL Staining

Paraffin sections were dewaxed using xylene and rehydrated through a series of ethanol gradients. TUNEL staining was performed using an apoptosis detection kit (Beyotime, #C1089) according to the manufacturer's instructions. The staining results were observed under a fluorescence microscope, and the apoptosis rate was calculated.

### Half‐Life of SPARC Measurement

3 × 10^5^ Caco‐2 cells were seeded in a 6‐well plate and either transfected with the YTHDF1 overexpression plasmids (Miaolingbio) or treated with the METTL3 inhibitor STM2457 (10 µM, MCE, USA, #2499663‐01‐1) for 48 h. Subsequently, the cells were exposed to cycloheximide (CHX, 40 µM, MCE, #66‐81‐9) for 0, 3, 6, 9, and 12 h. At each designated time point, the cells were harvested, and total protein lysates were prepared for western blot analysis to determine the expression levels of SPARC.

### Statistical Analysis

The statistical analysis in this study was performed using GraphPad Prism (version 8.0). Data were expressed as mean ± SD or SEM. Differences between groups were determined by student *t*‐test or one‐way analysis of variance. *p* value of <0.05 was considered statistically significant.

## Conflict of Interest

The authors declare no conflict of interest.

## Author Contributions

The authors checked and approved the final manuscript. T.S., W.C., and J.W. conceived and designed the experiments. J.W., and X.Z. performed most of the experiments and analyzed the data. Y.H. and Y.C. performed sample collection and clinical evaluation. J.Z., Z.D., and H.Z. performed a specific subset of the experiments and analyses. The manuscript was written by J.W. and revised by T.S., and W.C.

## Supporting information



Supporting Information

## Data Availability

The data that support the findings of this study are available from the corresponding author upon reasonable request.
