# Supplementary material for: Elevated SPARC Disrupts the Intestinal Barrier Integrity in Crohn's Disease by Interacting with OTUD4 and Activating the MYD88/NF‐κB Pathway
Source: Adv Sci (Weinh). 2025 Jan 30;12(11):2409419. doi: 10.1002/advs.202409419 (PMC11923920; doi:10.1002/advs.202409419)
Supplement: Supplementary file 1 — Supporting Information [file ADVS-12-2409419-s001.docx]

| **Table S1. Primers for genotype identification** | | |
| --- | --- | --- |
| **Primer Name** |  | **Primer sequence** |
| F1 |  | CTACATTTCAAACTGCCATTCCCT |
| R1 |  | CCAATTAGTTGTCCCCAGAACACA |
| R2 |  | CTGCTAGTCAAGCCTCAATACGTC |

| **Table S2. Primers for RT-qPCR** | | |
| --- | --- | --- |
| **Primer Name Primer sequence** | | |
| hsa-GAPDH-Forward |  | GGAGCGAGATCCCTCCAAAAT |
| hsa-GAPDH-Reverse |  | GGCTGTTGTCATACTTCTCATGG |
| hsa-SPARC-Forward |  | TGAGGTATCTGTGGGAGCTAATC |
| hsa-SPARC-Reverse |  | CCTTGCCGTGTTTGCAGTG |
| hsa-MLCK-Forward |  | CCCGAGGTTGTCTGGTTCAAA |
| hsa-MLCK-Reverse |  | GCAGGTGTACTTGGCATCGT |
| mus-GAPDH-Forward |  | AGGTCGGTGTGAACGGATTTG |
| mus-GAPDH-Reverse |  | GGGGTCGTTGATGGCAACA |
| mus-TNF-α-Forward |  | CAGGCGGTGCCTATGTCTC |
| mus-TNF-α-Reverse |  | CGATCACCCCGAAGTTCAGTAG |
| mus-IFN-γ-Forward |  | GCCACGGCACAGTCATTGA |
| mus-IFN-γ-Reverse |  | TGCTGATGGCCTGATTGTCTT |
| mus-IL1β-Forward |  | GAAATGCCACCTTTTGACAGTG |
| mus-IL1β-Reverse |  | TGGATGCTCTCATCAGGACAG |
| RIP-SPARC-Forward |  | TGAGGTATCTGTGGGAGCTAATC |
| RIP-SPARC-Reverse |  | CCTTGCCGTGTTTGCAGTG |
| MeRIP-SPARC-Forward |  | CCCATTGGCGAGTTTGAGAAG |
| MeRIP-SPARC-Reverse |  | CAAGGCCCGATGTAGTCCA |

| **Table S3. Target sequence of SPARC siRNA** | | |
| --- | --- | --- |
| **si-RNA** |  | **Primer sequence** |
| si-SPARC-1 |  | GGACAACAACCTTCTGACT |
| si-SPARC-2 |  | GACTTCGAGAAGAACTATA |
| si-SPARC-3 |  | TGTGCGAGCTGGATGAGAA |

**
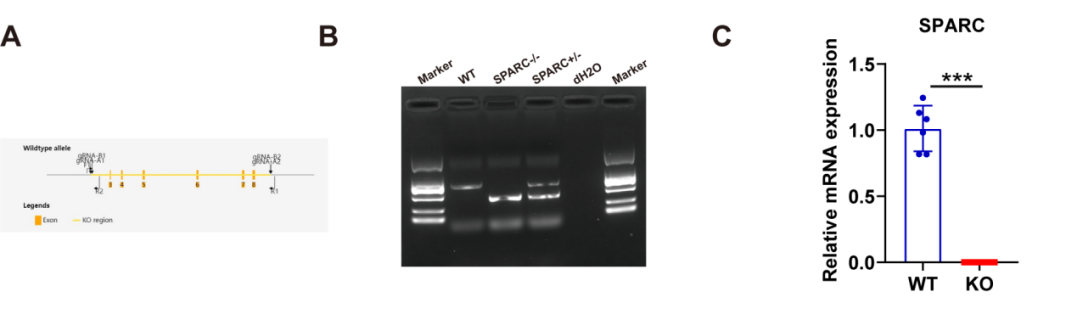
**

**Figure. S1** A) Schematic illustration of the strategy to generate SPARC KO mice. SgRNAs were designed to delete exons 3 to 8 of the SPARC gene. B) Genomic identification of SPARC KO mice. C) The mRNA expression of SPARC in SPARC KO mice (n=6) and WT mice (n=6).

**
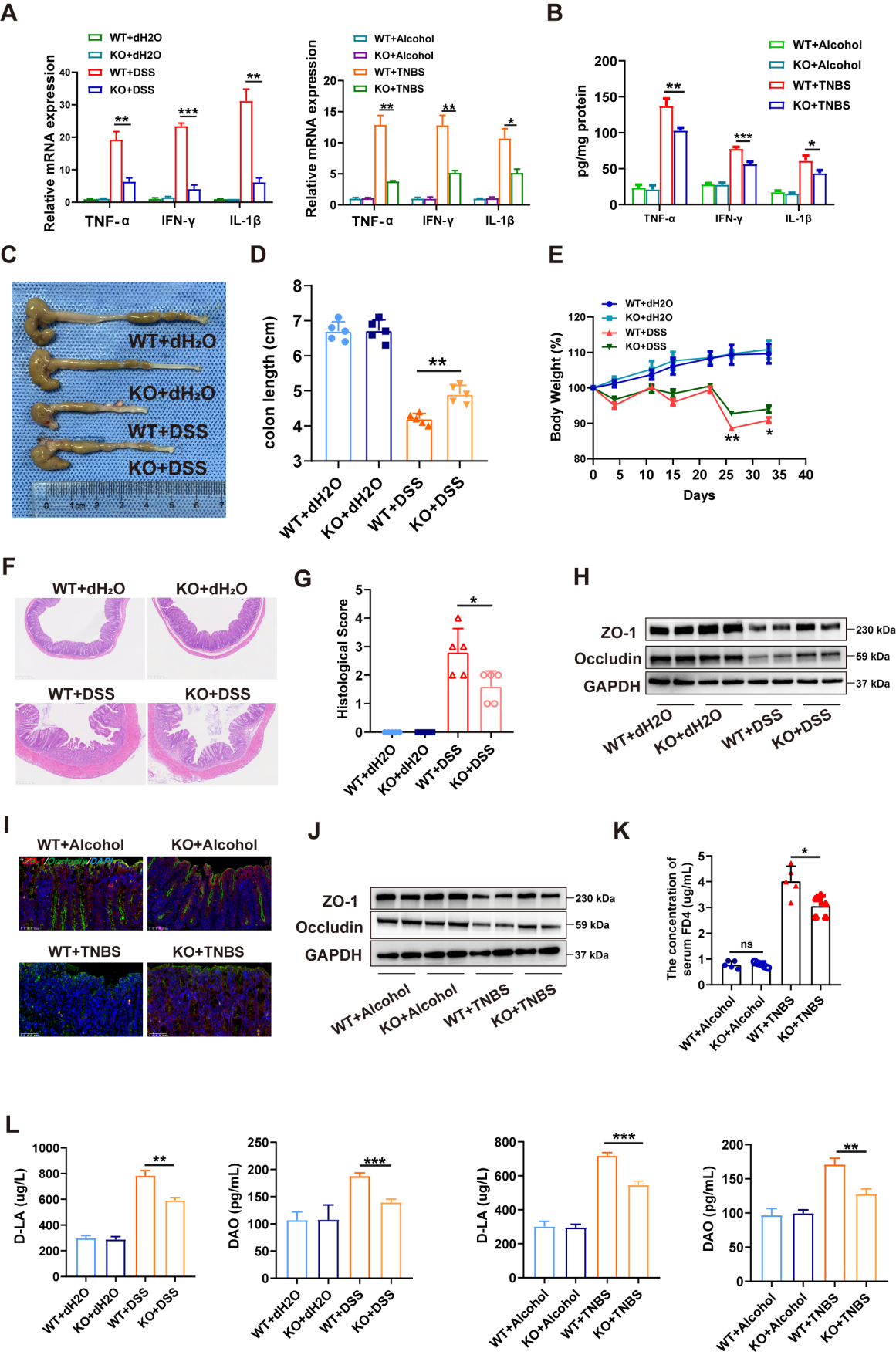
**

**Figure. S2** A) Relative mRNA levels of TNF-α, IFN-γ and IL-1β in the colon tissues were assessed by RT-qPCR. B) Relative protein levels of TNF-α, IFN-γ and IL-1β in the colon were assessed by ELISA. C-H) Chronic colitis model was constructed in SPARC knockout mice and WT mice. Body weight loss were measured(E). Sections of paraffin-embedded colon tissues were stained with H&E(F). (G)Histological scores of the colon of colitis mice. (H)The expression of TJ in the colon of chronic DSS mouse models was detected by western blotting. I- J) The expression of TJ in the colon of TNBS colitis mice was detected by immunofluorescence staining(I) and western blotting(J). Scale bars, 50 µm. K)The concentration of serum FD4. L)The contents of D-LA and DAO in serum of mice challenged with DSS or TNBS were determined by ELISA.


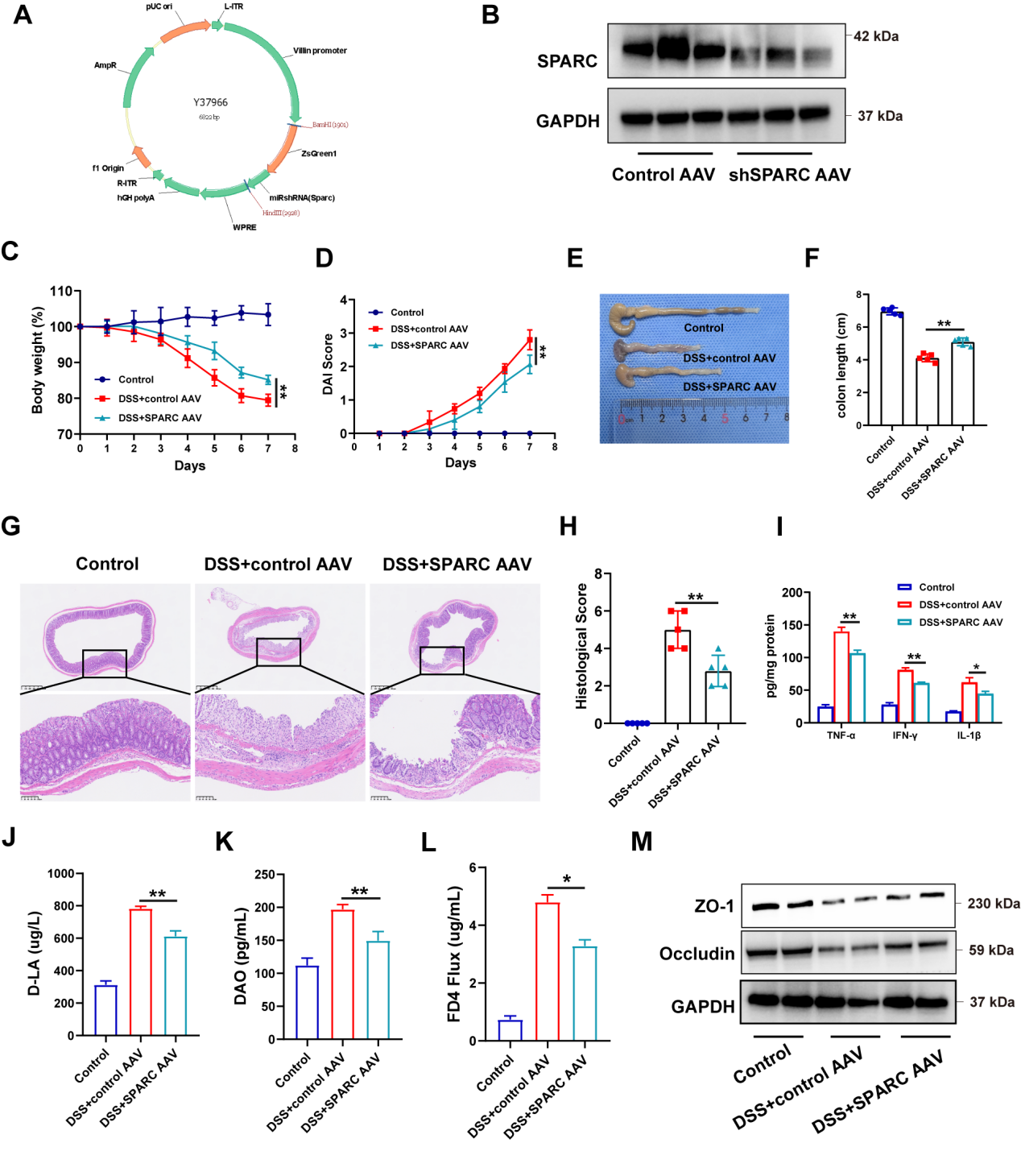


**Figure S3** A) Map of the vector of adeno-associated virus. B) The protein levels of SPARC in the colonic epithelium of mice in the control-AAV and shSPARC-AAV groups were determined by western blot. C-F) Body weight loss (C), the disease activity index (DAI) score (D), colon image (E) and colon length (F) were measured. G) Sections of paraffin-embedded colon tissues were stained with H&E. H) Histological scores of the colon of colitis mice. I) Relative protein levels of TNF-α, IFN-γ and IL-1β in the colon were assessed by ELISA. J and K) The contents of D-LA (J) and DAO (K) in serum of mice challenged with DSS were determined by ELISA. L) The concentration of serum FD4. M) The expression of TJ proteins in the colon of DSS colitis mice was detected by western blotting.

**
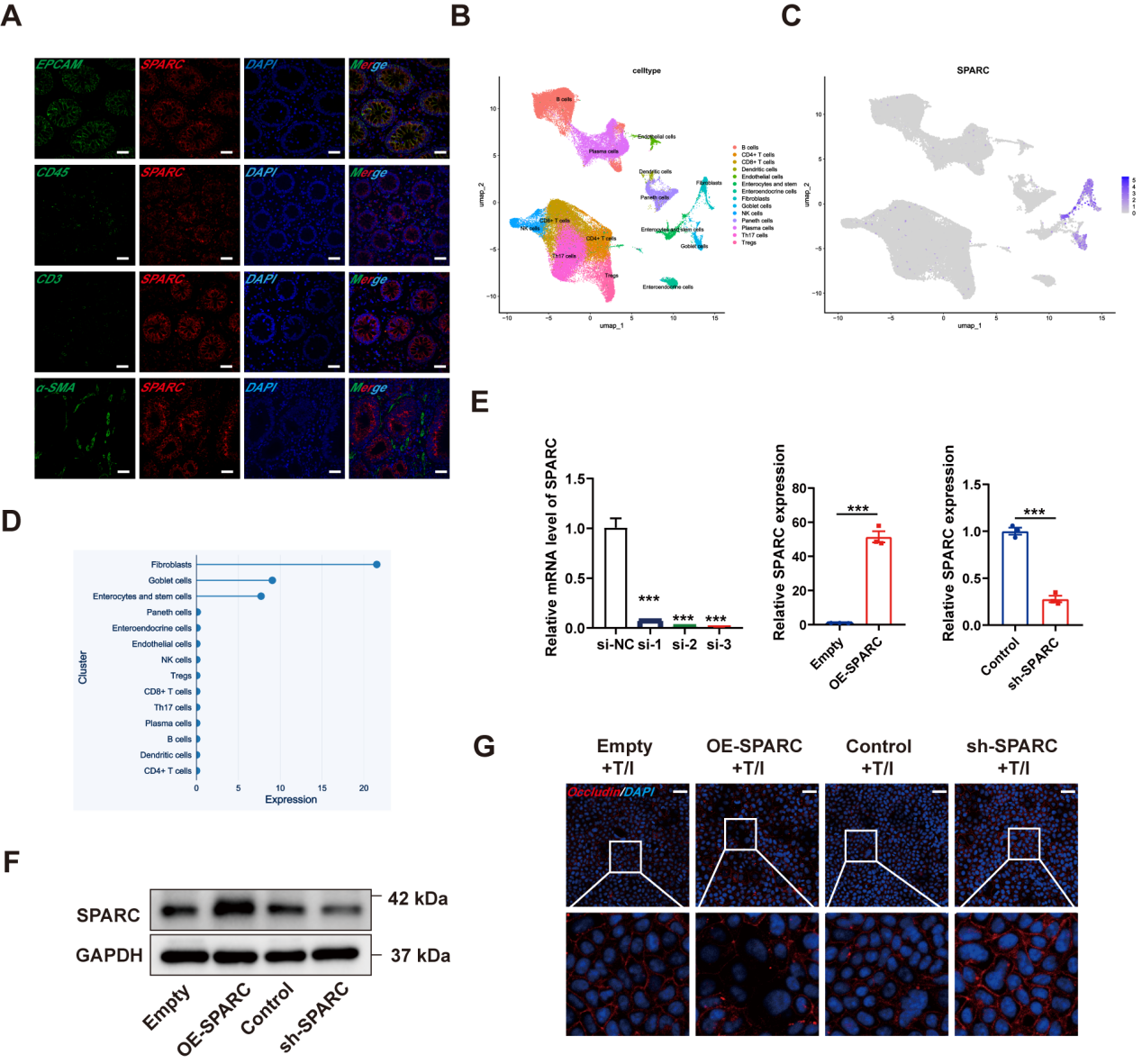
**

**Figure. S4** A) Immunofluorescence for SPARC was used to assess its cellular localization on colon sections from CD patients (red, SPARC; green, EPCAM/CD45/CD3/α-SMA; DAPI nuclear staining). Scale bars, 25 µm. B) The UMAP plot illustrates single cells colored by shared nearest neighbor clusters and cell types, derived from single-cell sequencing of human colonic biopsy samples in the GSE134809 dataset. C) The feature plot highlights SPARC expression levels, with each dot representing an individual cell. D)Relative mRNA expression of SPARC in diferent cell types in the GSE134809 dataset. E）The SPARC mRNA levels in Caco-2 cells transfected with the SPARC siRNAs were measured by RT-qPCR. F) The protein expression of SPARC in Caco-2 cells infected with sh-SPARC lentivirus or SPARC overexpressing lentivirus. G) Representative immunofluorescence images of occludin expression in SPARC overexpression or knockdown Caco-2 cells stimulated by TNF-α/IFN-γ. Scale bars, 50 µm.


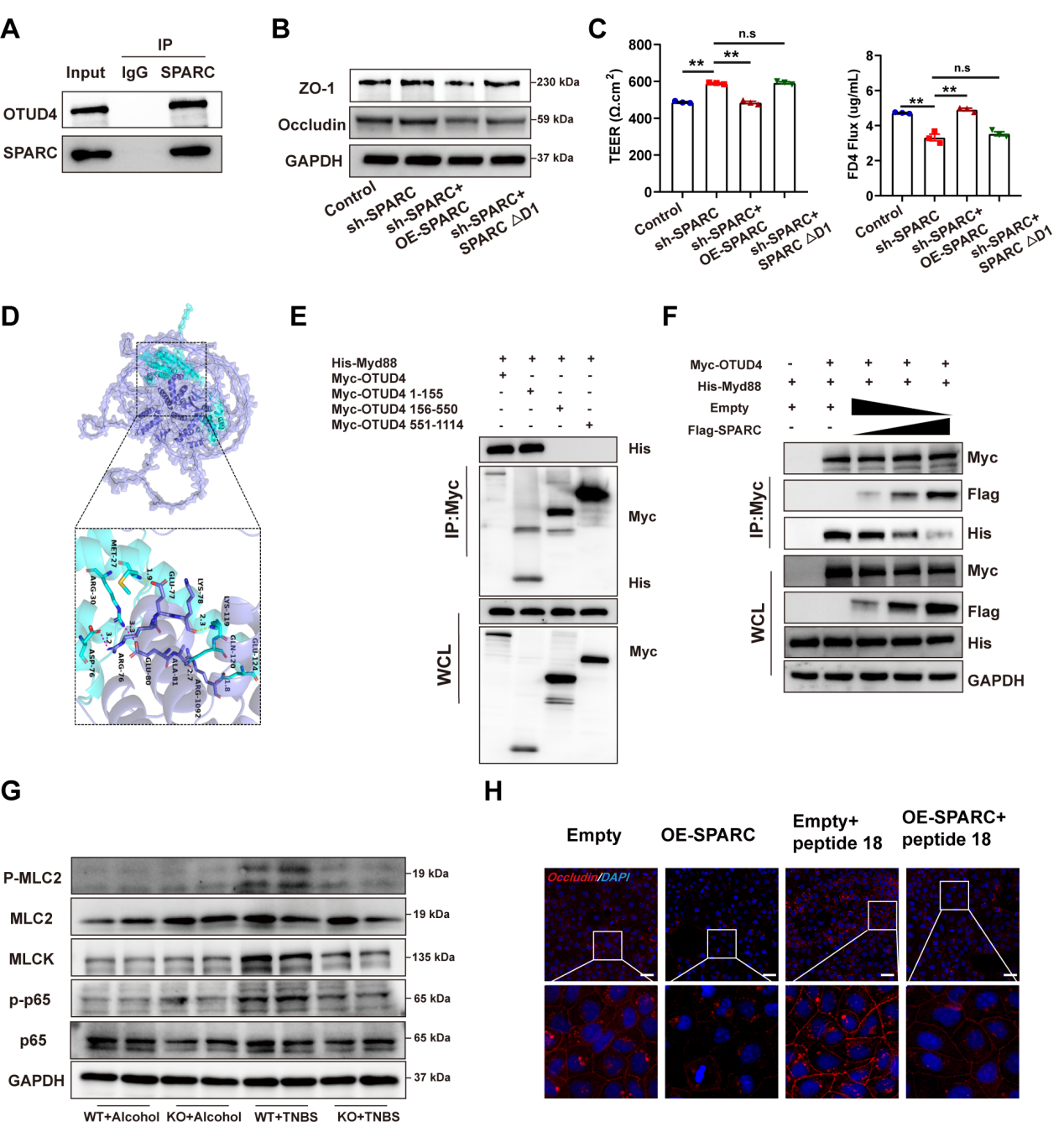


**Fig. S5** A) Co-IP analysis showed SPARC interacted with OTUD4 in DSS-induced colon. B).The protein expression of ZO-1 and occludin in various groups of Caco-2 cells stimulated by TNF-α/IFN-γ. C). TEER and FD4 permeability of Caco-2 monolayer cell model after TNF-α/IFN-γ stimulation. D) OTUD4 and MYD88 prediction structures were generated by Alphafold. OTUD4 was represented as a dark blue cartoon model. MYD88 was shown as a cyan cartoon model. Their joint points were shown as rod structures of the corresponding colors. E) Co-IP analysis showed the binding region between OTUD4 and MYD88. F) Myc-OTUD4, His-Myd88, and empty vector together with different concentrations of Flag-SPARC were co-overexpressed in HEK293T cells for 48 h, and competitive binding co-IP experiments were performed with anti-Myc antibody and analyzed with western blot. G) The protein expression of p65, p-p65, MLCK, MLC2 and p-MLC2 in colon tissues from WT or SPARC mice challenged with TNBS. H) The expression of Occludin in SPARC overexpression Caco-2 cells treated with Peptide 18 was detected by immunofluorescence. Representative images were shown. Scale bars, 50 µm.


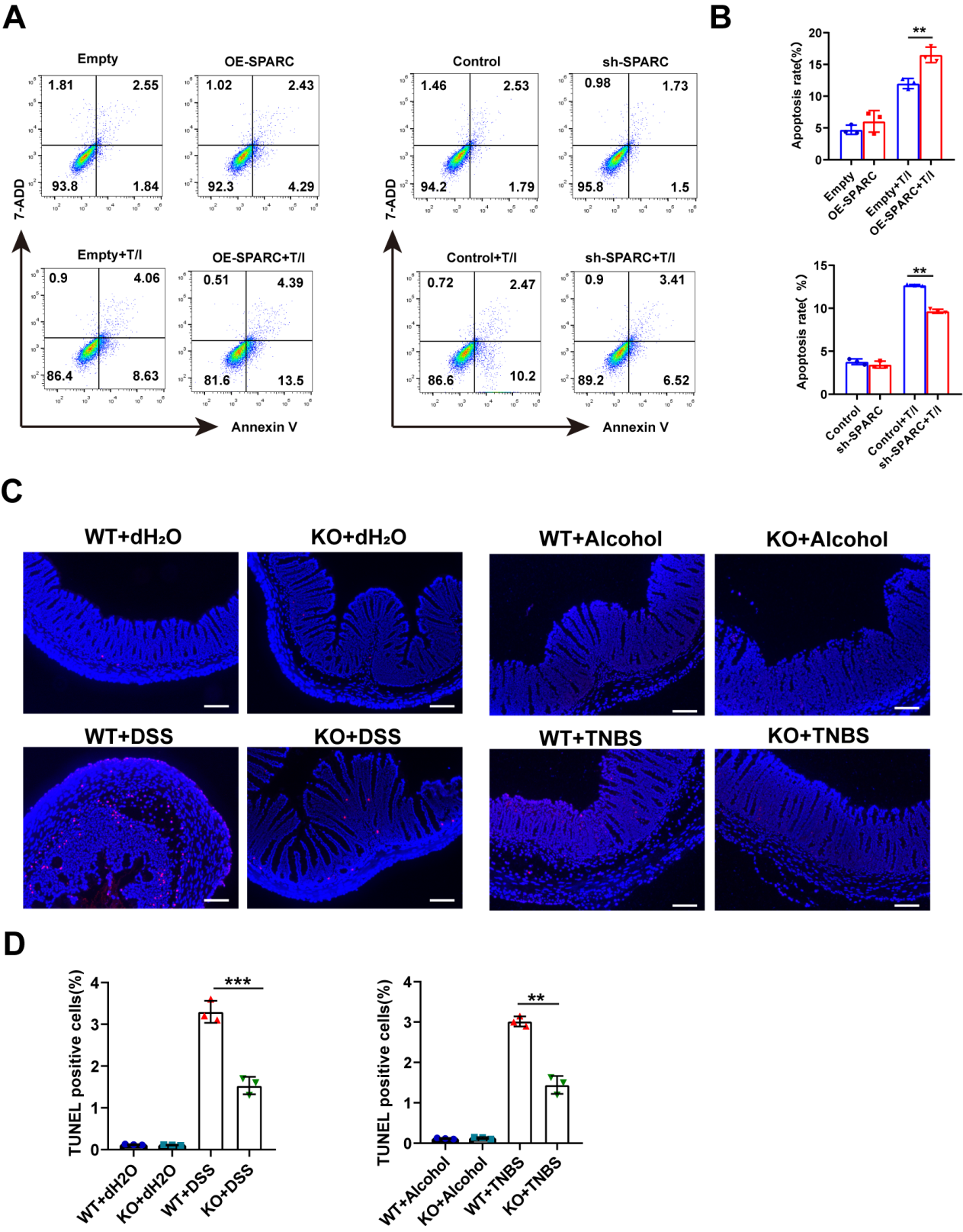


**Figure.S6** A-B) Flow cytometry was used to detect the apoptosis of SPARC overexpression or knockdown Caco-2 cells treated with TNF-α/IFN-γ (T/I). Representative cytofluorometric pictograms were shown(A). The quantitative summaries are shown in (B). C-D) TUNEL staining in colon tissues of WT or SPARC KO mice challenged with DSS or TNBS. Representative images were shown(C). Scale bars, 100 µm. The quantitative summaries are shown in (D).


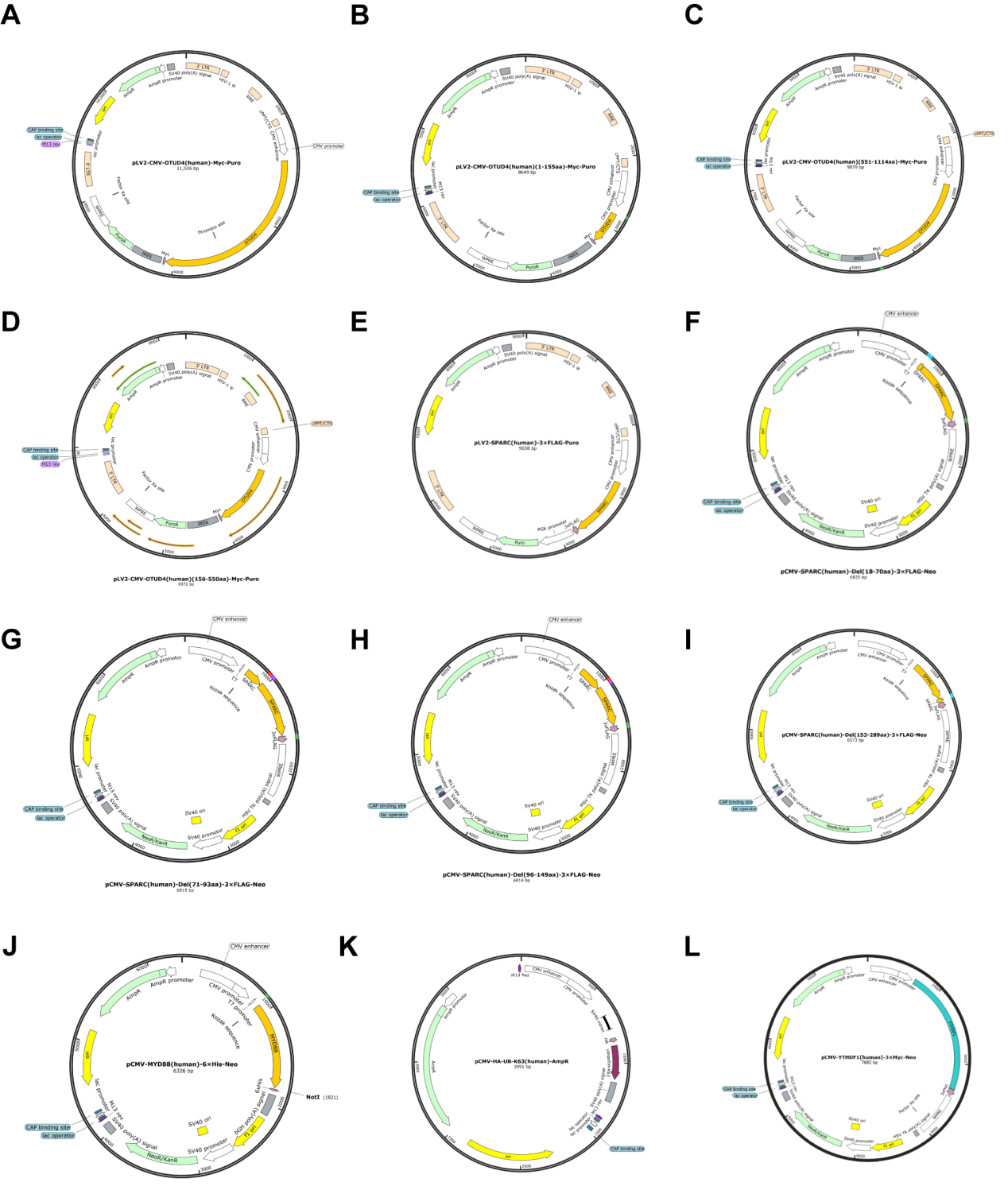


**Figure.S7** A)The plasmid map for Myc-OTUD4. B)The plasmid map for Myc-OTUD4(1-155aa). C)The plasmid map for Myc-OTUD4(551-1114aa). D)The plasmid map for Myc-OTUD4(156-550aa). E)The plasmid map for Flag-SPARC. F)The plasmid map for Flag-SPARC Δ1 (18-70). G)The plasmid map for Flag-SPARC Δ2 (71-93). H)The plasmid map for Flag-SPARC Δ3 (96-149). I)The plasmid map for Flag-SPARC Δ4 (153-289). J)The plasmid map for His-MYD88. K)The plasmid map for HA-Ub-K63. L)The plasmid map for Myc-YTHDF1.
